# A protocol of systematic review and meta-analysis of narrow band imaging endoscopy in detection of early gastric cancer

**DOI:** 10.1097/MD.0000000000021269

**Published:** 2020-07-17

**Authors:** Xiao-yu Liu, Jun Zhang

**Affiliations:** a Department of Endoscopy Center, Yulin No.2 Hospital, Yulin; b Department of Gastroenterology, Second Affiliated Hospital of Xi’an Jiaotong University School of Medicine, Xi’an, China.

**Keywords:** accuracy, diagnosis, early gastric cancer, narrow band imaging endoscopy

## Abstract

**Background:**

Although previous studies have utilized narrow band imaging endoscopy diagnosis (NBIED) in detection of patients with early gastric cancer (EGC), there are still inconsistent results. Thus, this study will explore the accuracy of NBIED in detection of patients with EGC.

**Methods:**

We propose to perform literature search of potential studies investigating the accuracy of NBIED in detection of patients with EGC in MEDLINE, EMBASE, Cochrane Library, Web of Science, WANGFANG, VIP database and China National Knowledge Infrastructure from the beginning of each database to January 31, 2020 without restrictions to language and publication time. Two authors will independently scrutinize these databases to identify studies that satisfy all predefined eligibility criteria. We will check study quality and analyze outcome data using Quality Assessment of Diagnostic Accuracy Studies tool, and RevMan 5.3 software respectively.

**Results:**

We anticipate the results of this study will afford additional insight into the appraising of the accuracy of NBIED in patients with EGC.

**Conclusion:**

The findings of this study will be useful informing diagnostic decisions for the diagnosis of patients with EGC.

PROSPERO registration number: PROSPERO CRD42020171053.

## 1 Introduction

Gastric cancer is 1 of the most common malignant cancers, and also the leading causes of cancer deaths worldwide,^[1–5]^ which manifests as abdominal pain and indigestion.^[3,6]^ It is reported that 1,033,701 new cases and 782,685 deaths were detected in 2018 around the world.^[7]^ Its high mortality rate is reported due to the fact that it is difficult to detect at early stage because it is typically asymptomatic.^[8,9]^ Thus, early detection is very essential to decrease its mortality.^[10–12]^

Studies suggested that narrow band imaging endoscopy diagnosis (NBIED) is reported in detection of patients with early gastric cancer (EGC).^[13–30]^ However, no systematic review has addressed it, and inconsistent conclusions are drawn based on the individual study. Therefore, the present study is designed to synthesize the presently available evidence to investigate the accuracy of NBIED in detection of patients with EGC.

## 2 Methods

### 2.1 Objective

This study will aim to explore the accuracy of NBIED in detection of patients with EGC.

### 2.2 Study registration

This study was funded and registered on PROSPERO with CRD42020171053. We have reported this study based on the guideline of Preferred Reporting Items for Systematic Reviews and Meta-Analysis Protocol statement.^[31]^

### 2.3 Inclusion criteria for study selection

#### 2.3.1 Type of studies

All potential case-controlled studies (CCSs) reporting the accuracy of NBIED in detection of patients with EGC will be included in this study. We will exclude any other studies, such as animal studies, review, and comment.

#### 2.3.2 Type of participants

In this study, the reports of all potential participants with histological-proven EGC will be included, in spite of their country, race, age, and gender.

#### 2.3.3 Type of index test

Index test: All studies used NBIED to detect EGC as their index test.

Reference test: All studies utilized histological-proven EGC alone as their reference test.

#### 2.3.4 Type of outcome measurements

Primary outcomes are sensitivity and specificity. Secondary outcomes are positive likelihood ratio, negative likelihood ratio, and diagnostic odds ratio.

### 2.4 Data sources and search strategy

#### 2.4.1 Electronic searches

We will propose a systematic and comprehensive literature search in MEDLINE, EMBASE, Cochrane Library, Web of Science, WANGFANG, VIP database and China National Knowledge Infrastructure from inception up to the January 31, 2020 without restrictions to language and publication time. We will create a search strategy sample for MEDLINE (Table 1), and will modify similar search strategies for other electronic databases.

**Table 1:**
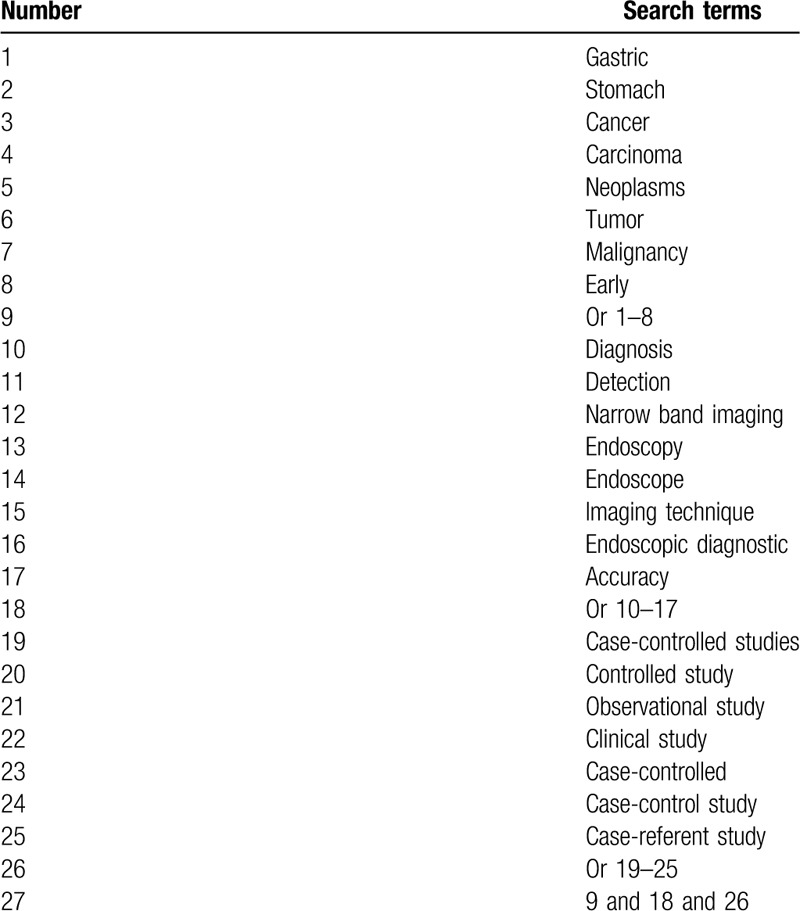
Search strategy applied in MEDLINE database.

#### 2.4.2 Other resources

Other resources will also be examined, such as Google Scholar, clinical trial registry websites, and reference list of relevant reviews.

### 2.5 Data collection and analysis

#### 2.5.1 Selection of studies

All records will be managed and duplicates will be eliminated using Endnote 7.0 software. Two authors will independently scan titles/abstracts of potential citations, and irrelevant records will be removed. Next, same 2 authors will obtain and review full-text of remaining articles against all inclusion criteria and will determine final eligible studies for suitability. Any divergences will be arbitrated by another author, and a consensus will be reached. We will demonstrate the process of study selection in a Preferred Reporting Items for Systematic Reviews and Meta-Analysis Protocol flowchart.

#### 2.5.2 Data collection and management

After study selection, all essential data will be collected from each eligible study by 2 independent authors using previous designed data extraction sheet. It includes first author, publication year, country, study setting, study design, age, race, sample size, number of true-positive, false-negative, true-negative, and false-positive, index and reference tests, and any related essential data. Any differences will be solved via discussion with a third author.

#### 2.5.3 Dealing with missing data

If unclear or missing data is identified, we will contact primary authors to obtain it. If it is not available, we will analyze data at hand using an intention-to-treat analysis.

### 2.6 Study quality assessment

Study quality of all eligible CCSs will be appraised by 2 independent authors using Quality Assessment of Diagnostic Accuracy Studies tool for each included study.^[32]^ It covers 4 aspects, and each 1 is assessed with risk of bias, judged by signaling questions. Any conflicts will be solved through discussion with the help of a third author.

### 2.7 Statistical analysis

#### 2.7.1 Data synthesis

RevMan V.5.3 and Stata V.12.0 softwares will be utilized to perform the descriptive analyses. We will display study results as descriptive statistics and 95% confidence intervals, and will draw receiver operating characteristic curves. The degree of statistical heterogeneity will be examined by *I*^*2*^ statistic. *I*^*2*^ ≤ 50% means homogeneity, while *I*^*2*^ > 50% suggests obvious heterogeneity. We will synthesize the data if eligible studies exert sufficient clinical homogeneity or if they are carried out in the same or comparable context. We will obtain pooled estimates of sensitivity, specificity, positive likelihood ratio, negative likelihood ratio, and diagnostic odds ratio. We will enter the data for 2 × 2 tables and will use bivariate model for meta-analysis. If necessary, a descriptive forest plot will be employed. If heterogeneity is identified as substantial, we will carry out a subgroup analysis. If it is still considerable after subgroup analysis, we will report study results as a narrative summary.

#### 2.7.2 Subgroup analysis

Subgroup analysis will be conducted to explore the sources of heterogeneity according to the differences in study information, and patient characteristics.

#### 2.7.3 Sensitivity analysis

Sensitivity analysis will be performed to determine the stability and robustness of study results by eliminating the low quality studies.

#### 2.7.4 Reporting bias

If a sufficient number of studies are included, we will examine reporting bias through funnel plots.^[33]^

### 2.8 Ethics and dissemination

This study does not need research ethic, because it will not use individual patient data. The results of this study will be published on a peer-reviewed journal or a related conference meeting.

## 3 Discussion

Previous studies explored the accuracy of NBIED in detection of patients with EGC.^[13–30]^ However, no systematic review has assessed the accuracy of NBIED in detection of patients with EGC. To our best knowledge, this will be the first systematic review focusing on this subject. It will provide evidence to assess the clinical value of NBIED in the detection of EGC. Its findings may benefit clinical practice and future studies. However, it may still suffer from several limitations: the methodological quality of eligible trials may be poor; the number of eligible trials may be insufficient, and the sample size of the included may be small.

## Author contributions

**Conceptualization:** Xiao-yu Liu, Jun Zhang.

**Data curation:** Xiao-yu Liu, Jun Zhang.

**Formal analysis:** Xiao-yu Liu, Jun Zhang.

**Funding acquisition:** Jun Zhang.

**Investigation:** Jun Zhang.

**Methodology:** Xiao-yu Liu.

**Project administration:** Jun Zhang.

**Resources:** Xiao-yu Liu, Jun Zhang.

**Software:** Xiao-yu Liu.

**Supervision:** Jun Zhang.

**Validation:** Xiao-yu Liu, Jun Zhang.

**Visualization:** Xiao-yu Liu, Jun Zhang.

**Writing – original draft:** Xiao-yu Liu, Jun Zhang.

**Writing – review & editing:** Xiao-yu Liu, Jun Zhang.
